# Feature Selection for Longitudinal Data by Using Sign Averages to Summarize Gene Expression Values over Time

**DOI:** 10.1155/2019/1724898

**Published:** 2019-03-19

**Authors:** Suyan Tian, Chi Wang

**Affiliations:** ^1^Division of Clinical Research, The First Hospital of Jilin University, 71 Xinmin Street, Changchun, Jilin 130021, China; ^2^Center for Applied Statistical Research, School of Mathematics, Jilin University, 2699 Qianjin Street, Changchun, Jilin 130012, China; ^3^Department of Biostatistics, College of Public Health, University of Kentucky, Lexington, KY 40536, USA; ^4^Markey Cancer Center, University of Kentucky, 800 Rose St., Lexington, KY 40536, USA

## Abstract

With the rapid evolution of high-throughput technologies, time series/longitudinal high-throughput experiments have become possible and affordable. However, the development of statistical methods dealing with gene expression profiles across time points has not kept up with the explosion of such data. The feature selection process is of critical importance for longitudinal microarray data. In this study, we proposed aggregating a gene's expression values across time into a single value using the sign average method, thereby degrading a longitudinal feature selection process into a classic one. Regularized logistic regression models with pseudogenes (i.e., the sign average of genes across time as predictors) were then optimized by either the coordinate descent method or the threshold gradient descent regularization method. By applying the proposed methods to simulated data and a traumatic injury dataset, we have demonstrated that the proposed methods, especially for the combination of sign average and threshold gradient descent regularization, outperform other competitive algorithms. To conclude, the proposed methods are highly recommended for studies with the objective of carrying out feature selection for longitudinal gene expression data.

## 1. Introduction 

Feature selection, a mighty tool to tackle the high dimensionality issue accompanying high-throughput experiments where the number of measured features (e.g., genes or metabolites), is much larger than that of samples and has been employed with increasing frequency in many research areas, including biomedical research. The ultimate goal of feature selection is to correctly identify features associated with the phenotypes of interest while ruling out irrelevant features as much as possible.

Because biological systems or processes are dynamic, it is useful for researchers to investigate gene expression patterns across time in order to capture biologically meaningful dynamic changes. With the rapid evolution of high-throughput technology, time series/longitudinal microarray experiments have become possible and even affordable. However, development of specific statistical methods dealing with expression profiles across time points has not kept pace.

One commonly used strategy is to stratify time series data into separate time points and then analyze these points separately. This approach may lead to inefficiency in statistical power by ignoring the highly correlated structure of gene expression values across time and thus result in failure to detect patterns of change across time [1–3].

An alternative strategy to conduct feature selection for longitudinal gene expression data is to use statistical methods capable of detecting different expression patterns across time between groups. Examples include Significance Analysis of Microarray [4], Extraction of Differential Gene Expression (EDGE) [1, 5], Linear Models for Microarray Data (limma) [6], and Microarray Significant profiles [7]. EDGE uses a spline approach and is one of the first methods to specifically address identification of differentially expressed genes across time [8]. In contrast, the limma method has a more general purpose and is easily understood and implemented [7]; therefore, it has gained extreme popularity and become the gold standard to detect differentially expressed genes under different scenarios (e.g., two-group or multiple-group comparison) for microarray data. Nevertheless, because the limma method usually does not correctly account for the order of time points or the correlation structure introduced by multiple observations from the same subject, it tends to be outperformed by other relevant methods. Since these statistical strategies usually screen genes one by one according to the magnitude of a gene's relevance to the phenotype of interest, they may be classified as the filter methods [9]. The big drawback of filter methods is that many false positive genes remain in the final model [9].

Some researchers have extended two typical longitudinal data analysis strategies, namely, the generalized estimating equation (GEE) method [10] and a mixed model [11], to carry out feature selection for time series gene expression profiles. The GEE-based screening procedure [3], penalized-GEE (PGEE) [2], and glmmLasso [12] methods belong to this category. Among them, the GEE-based screening procedure fits a GEE model to each gene and then filters out the nonsignificant genes. By filtering genes one by one, this procedure is very likely to mistakenly include redundant genes highly correlated with the true relevant genes in the final gene list. The PGEE algorithm [2] adds the SCAD penalty term [13] to the corresponding quasilikelihood function of a GEE model to implement feature selection and model construction. In contrast, the glmmLasso method [12] maximizes the corresponding penalized log likelihood function of a generalized linear mixed model using a combination of the gradient ascent method with the Fisher scoring algorithm in order to realize the selection of relevant genes for longitudinal data and the estimation of their coefficients simultaneously. Although the PGEE method and the glmmLasso method can carry out feature selection for longitudinal expression data and also eliminate or alleviate the inefficiency caused by separate analysis at each time point, these methods cannot handle extremely large numbers of genes [2, 14], which are often encountered in longitudinal gene expression profiles. For a selective review of methods capable of carrying out feature selection for longitudinal omics data, see Albrecht et al. [8].

A gene set or pathway refers to a set of genes that are highly likely to coregulate/coexpress to influence a biological process (examples are gene sets defined in the Gene Ontology project [15] or Chaussabel's functional modules [16]). According to this definition, one specific gene's expression values collected over multiple time points may be regarded as a gene set, rendering the scores at pathway/gene set level sound options to summarize a gene's expression values at different time points into a single value. Thus, a reasonable alternative way of dealing with time series gene expression data is to use those pathway-level summary scores. Popular choices of a summary score include the means [17], medians or first principal components (PC) of time-course gene expression values [18], or the pathway deregulation scores proposed by Drier et al. [19]. Unfortunately, all these summary scores have major drawbacks. For instance, when a gene exhibits opposite association with the phenotype of interest at different time points, the mean average operator that does not account for effect directions may cancel out the different time effects of this gene [20]. In contrast, construction of pathway deregulation scores is more theoretically complicated and computationally intensive, requiring involvement of an expert statistician. For the first principal component summary, it is well known that the direction of the largest variance of the gene expression values is pinpointed instead of the genes that are most related to the phenotypes of interest.

The sign average [21, 22], also known as the Direction Aware Average [20], takes into account the directions of association between genes and the phenotype of interest as well as genes' expression values and might be less subject to overfitting since these directions are more robust than their estimated effects [20, 21]. As opposed to the average operator, the sign average considers not only the expression values of a gene at each time point but also the direction of its association with the phenotype of interest at those time points; therefore, positive and negative associations do not cancel each other out. The sign average is an average in essence, however, and not as sophisticated as the pathway deregulation scores. Given the fact that the sign average is capable of mitigating these two drawbacks simultaneously, it may be a more suitable choice to summarize a genes' expression value at the gene set level or a gene's expression values over time.

In this study, we consider a scenario that has a long history—traumatic injury with subsequent infection. In ancient times, traumatic injury with subsequent infection was a common cause of death. Even today, massive injury remains life-threatening in many developed countries [23, 24]. In a clinical study carried out recently [25], patients with traumatic injury were classified into those experiencing uncomplicated recovery and those with complicated recovery based on the duration of recovery. Specifically, uncomplicated recovery was defined as recovery within 5 days versus complicated recovery, which was defined as recovery after 14 days, no recovery by 28 days, or death. In subsequent studies, Xiao et al. [25] and Zhang et al. [25, 26] questioned whether a different expression pattern occurs across time in the two extreme scenarios of clinical recovery. Xiao et al. [25] used the EDGE method [1, 5] to examine the corresponding longitudinal expression profiles. We propose a procedure to identify discriminative genes for longitudinal data; in other words, using the sign average method to generate a pseudogene to represent a specific gene's expression values over time. A classic feature selection method can then be applied using the pseudogenes as predictors to identify a gene signature for segmentation of complicated recovery and uncomplicated recovery.

## 2. Methods and Materials

### 2.1. Experimental Data

Raw data were downloaded from the Gene Expression Omnibus database (GEO: http://www.ncbi.nlm.nih.gov/geo/; accession number GSE36809) and hybridized on Affymetrix HGU133 plus2 chips. The data included 167 severe blunt trauma patients. In this study, only patients with uncomplicated recovery (within 5 days) and patients with complicated recovery (recovery after 14 days, no recovery by 28 days, or death) were considered.

We refined our inclusion criteria by limiting the uncomplicated group to patients who had data at 0-5 time points and the complicated group to those who had data at more than 5 time points. Further, because the longest follow-up is 14 days for patients without complication, we truncated the data for patients with complication to 14 days as well. The time points under consideration were days 1/2, 1, 4, 7, and 14. In total, we included 97 patients: 55 experiencing uncomplicated recovery and 42 having complicated recovery.

Next, our dataset (n=97) was divided randomly into two subsets with a ratio of 3:2. The resulting datasets served as the training set and the test set, respectively.

### 2.2. Pre-Processing Procedures

Raw data (CEL files) of the microarray data set were downloaded from the GEO repository. Expression values were obtained using the fRMA algorithm [27] and were normalized using quantile normalization and then log2 transformed. For multiple probe sets matched to one specific gene, the one with the largest absolute log fold change was retained.

### 2.3. Statistical Methods

#### 2.3.1. Sign Average

To determine the directions of association using the sign average method, we compared each gene's expression value at each time point for the patients with complicated recoveries versus those with uncomplicated recoveries. Specifically, using the uncomplicated group as the reference, for patient i, gene k, at time point t, the corresponding gene expression X_ikt_  can be written as

(1) Xikt=β0kt+β1ktIpatient  i  in  the  complicated  group+εikt

Here, *ε*_*ikt*_ is the error term with a mean of 0 and a standard deviation of 1; I(x) is an indicator function whose value is 1 if the condition x is true and 0 otherwise. *β*_0*kt*_ represents the mean expression value of gene k at time point t for the uncomplicated patients; *β*_1*kt*_ represents the mean difference of gene k at time point t between the complicated patients and the uncomplicated patients.

At each time point for each gene a moderated t-test was fitted to decide if the specific gene is upregulated or downregulated for the complicated group against the uncomplicated group according to the sign of its estimated *β*_1kt_. Then different time points of a gene were stratified into either upregulated group U or downregulated group D. The upregulated group includes the time points for which increased expression is associated with a higher probability of experiencing complicated recovery (i.e., time points with positive *β*_1kt_ values). In contrast, the downregulated group includes the time points for which an increment in the gene's expression is associated with a lower probability for complicated recovery (i.e., the time points with negative *β*_1kt_ values).

Denoting the number of time points as |t_i_| for patient i (i=1,2,.. n), the sign average of a specific gene k over all measured time points for patient i is defined as

(2) $$\begin{array}{c} {Sign\;\;Average}_{ik}=\overset{\left|t_{i}\right|}{\underset{t=1}{\sum }}sign\left(\;\hat{\beta_{1kt}}\;\right)\times \frac{x_{ikt}}{\left|t_{i}\right|} \end{array}$$

A subscript i is used to indicate the time points measured for different patients and

(3) $$\begin{array}{c} sign\left(\;x\;\right)=\left\{\begin{array}{cc} 1 & if\;\;x>0\\ 0 & if\;\;x=0\\ -1 & if\;\;x<0 \end{array}\right. \end{array}$$

To put it simply, the sign average sums up a specific gene's expression values at all upregulated time points (i.e., U_k_) and the expression values at all downregulated time points (i.e., D_k_), separately. Then it takes the difference between these two summations and divides this difference by the number of time points measured. Obviously, the sign average also takes into account the directions of associations with the phenotype of interest.

Using a summary value to represent one gene's expression values across time makes all conventional feature selection algorithms applicable to longitudinal microarray data and also avoids the imbalance of observations in both groups (e.g., patients with uncomplicated recovery have five measures at most while patients with complicated recovery generally have more than five measures). Traditional methods such as a t-test are incapable of dealing with cases that have more than one observation from a group at a specific time point.

#### 2.3.2. Coordinate Descent (Optimizer)

The coordinate descent (CD) method [28] optimizes an objective function with respect to a single feature each time, iteratively cycling through all features until convergence. Given that CD has a linearly increased computing burden with the number of genes, it presents excellent power to optimize penalized regression problems. The CD method has been widely utilized in many studies [29–31]. Its key component is the soft-threshold operator S(x, y) defined below. This operator determines whose beta coefficients will deviate from zero, meaning the corresponding genes will be selected. Friedman et al. [28] provides a detailed description of the CD method.

(4) $$\begin{array}{c} S\left(\;x,y\;\right)=\left\{\begin{array}{cc} x-y & if\;\;x>0\;\;and\;\;y<\left|x\right|\\ x+y & if\;\;x<0\;\;and\;\;y<\left|x\right|\\ 0 & if\;\;y\geq \left|x\right| \end{array}\right. \end{array}$$

**Figure 1:**
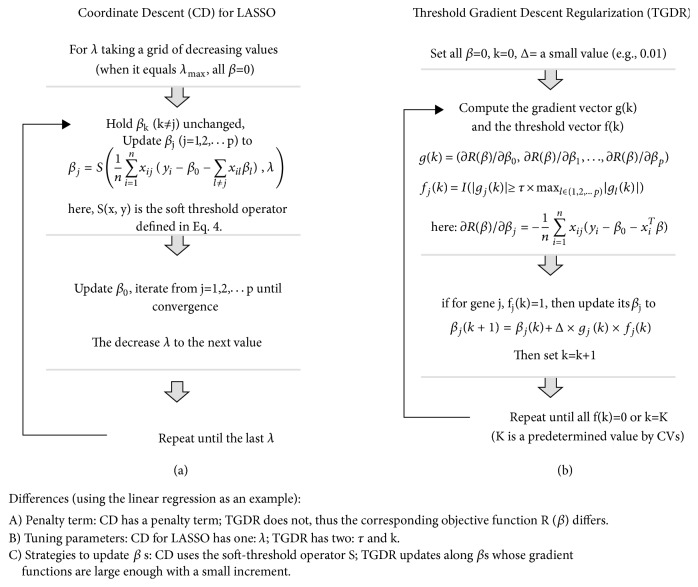
Comparison of methods for optimizing a penalized linear regression model. (a) Coordinate Descent. (b) Threshold Gradient Descent Regularization.

Figure 1(a) presents a flowchart of using CD to optimize a penalized linear regression with the LASSO penalty [32]. In the LASSO method, for the standardization of gene expression values across samples to have a mean of 0 and a standard deviation of 1, x in S(x, y) is related to g_j_(*β*) – the derivative/gradient of the objective function with respective to the j^th^  *β* coefficient, and y is the tuning parameter *λ*, restricting the L-1 norm of these *β* coefficients to be smaller than it is.

In this study, a regularized logistic regression model with a LASSO penalty was used, and it was solved using the CD method in the R glmnet package [28].

#### 2.3.3. Threshold Gradient Descent Regularization

The threshold gradient descent regularization (TGDR) method proposed by Friedman and Popescu [33] was adopted by Ma et al. [34] as an embedded feature selection algorithm that can select relevant genes and estimate corresponding coefficients simultaneously. For the definition of an embedded algorithm, see the review article by Saeys et al. [9]. After a thorough reading of the original paper and deep exploration of the algorithm [35–37], we found that it can be used as an optimization strategy to solve a regularized regression function.

In contrast to the CD method, the selection of genes in the TGDR method is realized by a comparison between a gene's gradient with the largest absolute gradient using a threshold function f_j_(*β*),

(5) $$\begin{array}{c} f_{j}\left(\;\beta \;\right)=I\left(\;\left|\;g_{j}\left(\;\beta \;\right)\;\right|\geq \tau \times {max}_{l}\left|\;g_{l}\left(\;\beta \;\right)\;\right|\;\right) \end{array}$$

Here, I(x) is an indicator with a value of 1 if the condition x inside the parentheses holds and 0 otherwise. Figure 1(b) presents a flowchart of using TGDR to optimize a linear regression model. Ma et al. [34] presents a detailed description of the TDGR method. Friedman [33] and Ma et al. [34] pointed out that when the gradient threshold *τ* in TGDR is fixed at 1, the TGDR algorithm provides a penalty approximately comparable to the LASSO term and a value of 0 corresponds to the ridge penalty. Major differences between the CD and the TGDR methods are presented in Figure 1.

In the current study, we fixed the tuning parameter *τ* at 1, which approximately corresponds to the LASSO model, and then we applied the TGDR method to the training set to obtain discriminative signatures. Two sets of signatures were compared to evaluate the pros and cons of the CD method versus the TGDR method. The R codes adapted from the programming of the meta-TGDR algorithm [38], which is an extension of the TGDR method to identify consistent relevant genes across multiple microarray studies, were used to implement the TGDR method.

#### 2.3.4. Performance Statistics

To evaluate the predictive performance of a classifier we used three metrics: Belief Confusion Metric (BCM), Area under the Precision-Recall Curve (AUPR), and misclassified error rate. Our two previous studies [39, 40] and the references therein describe these metrics in detail. Briefly, error rate = (false positives + false negatives)/(sample size) and captures the ability of correctly classifying the samples into their appropriate class. BCM captures the average confidence that a sample belongs in class k when it indeed belongs in that class. AUPR is computed as the average of the AUPR_k_ for each class and it captures the ability of correctly ranking the samples known to belong in a given class. The three metrics each range from 0 to 1. For BCM and AUPR, the closer to 1, the better a classifier is. The opposite is true for misclassified error rate.

Besides the discriminative/predictive performance, stability/reproducibility is of crucial importance for a gene signature as well [41]. Good stability does not guarantee a good predictive performance and true biomarker selection. On the other hand, if gene lists obtained from different training sets for the same disease share limited or no overlap at all, the utilization of such a gene signature in practice is impossible. To evaluate the reproducibility of the resulting gene lists, the Rand index is calculated. With k applications of a method (e.g., the k runs in a k-fold cross-validation), there are k gene lists (i.e., gs_1_, gs_2_,…, gs_k_). Upon these gene lists, a Rand index is defined as

(6) $$\begin{array}{c} Rand=\frac{2}{k\left(k-1\right)}\overset{k-1}{\underset{i=1}{\sum }}\;\overset{k}{\underset{j=i+1}{\sum }}\frac{\left|\cap \left({gs}_{i},{gs}_{j}\right)\right|}{\left|\cup \left({gs}_{i},{gs}_{j}\right)\right|} \end{array}$$

where ∩ represents the intersection between two gene lists, ∪ represents the union between the gene sets gs_i_ and gs_j_, and |  | represents the size of the gene set. As mentioned in our previous study [39], the optimal absolute values of these performance metrics vary from application to application. Therefore, the relative increase of those metrics obtained by an algorithm compared to another algorithm should be the focus.

### 2.4. Statistical Language and Packages

Statistical analysis was conducted in R, language version 3.3 (www.r-project.org). The R codes for the TGDR method and the sign average method are provided in the Supplementary File 1.

## 3. Results and Discussion

### 3.1. Real Data

#### 3.1.1. Validation

After randomly dividing our data into two sets (one serving as the training set and the other as the test set), the sign averages for genes under consideration in the training set were calculated. A 5-fold cross validation was used to decide the optimal value for the tuning parameter in the coordinate descent method or the threshold gradient descent regularization method.

#### 3.1.2. Selecting Relevant Genes

**Figure 2:**
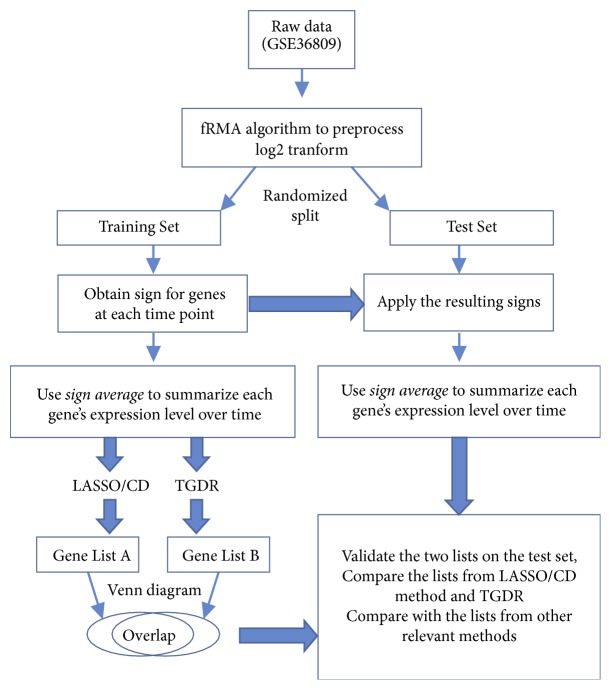
Study schema of the injury data application.

Briefly, the training set was divided into 5 roughly equal-sized subsets in which the ratio of complicated recovery to uncomplicated recovery was approximately the same as that of the whole training set. For 4 of the subsets, the LASSO/CD method (LASSO is the penalty function considered and CD is the optimization method) and the TGDR method were applied to select relevant genes and estimate their corresponding coefficients. The misclassified cases were counted by validating the resulting classifier to the remaining subset. This process was repeated 5 times with the five respective subsets serving as the test set only once. The misclassified errors were then aggregated for the whole training set. The optimal cutoff of the tuning parameter was the one having the smallest misclassified error. Using the optimal value of the tuning parameter, a final model was obtained using the training set and then was validated on the test set. The study schema is given in Figure 2, and the proposed methods are abbreviated as the sign average and LASSO/CD method and the sign average and TGDR method, respectively.

To evaluate the proposed method more comprehensively, we applied several relevant methods, i.e., EDGE [5], limma [6], glmmLASSO [12], LASSO [32], and TGDR [33] separately for each time point. For the last two methods, a subject's membership was determined using the average posterior probabilities, i.e., the means of calculated posterior probabilities at individual time points. For the limma and EDGE methods, an additional linear support vector machine model was fitted to calculate the posterior probabilities given that these three methods are only able to identify potentially relevant genes.  Table 1 provides an overview of the methods considered in this study.

#### 3.1.3. Predictive Capacity

The results are presented in Table 2. Based on the performance statistics under consideration (i.e., BCM, AUPR, misclassified error rate, and the Rand Index), these methods were divided into roughly three categories with decreasing performance. The two proposed methods belong to the first stratum; limma, EDGE, and simple SAMGSR belong to the second stratum; and the separate LASSO/TGDR method as well as the glmmLASSO method belongs to the last stratum. Specifically, regarding the predictive capacity, both proposed methods are ranked as the first two methods, with the sign average and TGDR method having an error rate of 35.1%, a BCM of 0.59, and an AUPR of 0.662 and the sign average and LASSO/CD method having an error rate of 37.8%, a BCM of 0.605, and an AUPR of 0.626, respectively. On the other hand, the limma method has very good stability but its predictive performance is slightly inferior to the two proposed methods. Although the glmmLASSO method outperforms the sign average and TGDR method in having the best model stability, its predictive performance on the test set is only better than that of the separate LASSO method, which drags its overall performance down. Additionally, when the tuning parameter *λ* is set as a value smaller than 15, the glmmLASSO algorithm crashes. This makes us suspect that similar to the PGEE method [2], the glmmLASSO algorithm also encounters difficulty in tackling extremely high dimensionality issues. Further investigation is warranted. To conclude, the sign average and TGDR method has the best overall performance versus other competitive methods.

To explore whether the sign average method provides a good summary of expression values across time points, we also considered other scores (means, medians, and first principal components) for individual gene expression values and combined those scores with the LASSO/CD or TGDR method to train the final models. The results are provided in Table 2. As expected, the sign average has the lowest error rate, the highest BCM, and AUCR and thus is superior to other summary scores regarding these performance statistics. This is because the sign average considers both the expression value and the directions of association with the phenotype of interest at individual time points. In contrast, the median may only consider a gene's expression value at a specific time point (the specific time point may vary for different samples, where the direction of association may also differ). The mean score only considers expression values, leading to some degree of cancellation between a positive association and a negative association. The first PC score only considers the factor/PC that explains the most variance among expression values over time, thus taking into account the least useful information for the classification problems.

#### 3.1.4. Relevance of Genes Identified by TGDR or LASSO/CD

Next, we focused on the unique genes identified by either the sign average and LASSO/CD method or the sign average and TGDR method and explored the biological relevance of these genes. According to the Genecards database (www.genecards.org), out of the five unique genes identified by TGDR, only DPYD, NFE2L2, and TLR5 are directly related to injury, whereas only TNFSF10 presents such a direct relation among the 7 unique CD genes. Although none of these 12 genes are indicated by the Genecards database to be directly related to* traumatic* injury, DPYD, NFE2L2, TLR5, and TLR8 of the TGDR unique genes are* indirectly* related to traumatic injury, whereas 5 of the CD unique genes (PPP2CB, TNFSF10, LGALS2, IGSF6, and PUS3) are indirectly related. Among the 4 unique TGDR genes indirectly related to traumatic injury, the Genecards database [42] summarizes that both TLR5 and TLR8 encode members of the toll-like receptor (TLR) family, which plays a fundamental role in pathogen recognition and activation of innate immune responses. These receptors recognize distinct pathogen-associated molecular patterns that are expressed on infectious agents. NFE2L2 (Nuclear Factor, Erythroid 2 Like 2) encodes a transcription factor that regulates genes that contain antioxidant response elements (ARE) in their promoters; many of these genes encode proteins involved in response to injury and inflammation. In contrast, among the 5 unique CD genes indirectly related to traumatic injury, PPP2CB (Protein Phosphatase 2 Catalytic Subunit Beta) encodes the phosphatase 2A catalytic subunit. Protein phosphatase 2A is one of the four major Ser/Thr phosphatases, and it is implicated in the negative control of cell growth and division. The Genecards database [42] gives the remaining genes very low confidence scores on their relevance.

#### 3.1.5. Relevance of Genes Identified by Both TGDR and LASSO/CD

Finally, we explored the biological meaning of genes identified by both methods in the Genecards database. We found that 11 of these overlapped genes are directly related to injury while the rest of them are indirectly related to injury. Additionally, all of those genes are indirectly related to traumatic injury. Specifically, the protein encoded by A2M (Alpha-2-Macroglobulin) is a protease inhibitor and cytokine transporter. A2M uses a bait-and-trap mechanism to inhibit a broad spectrum of proteases including trypsin, thrombin and collagenase. It can also inhibit inflammatory cytokines, and therefore disrupt inflammatory cascades. SPP1 (Secreted Phosphoprotein 1) encodes a protein that binds tightly to hydroxyapatite and acts as a cytokine involved in enhancing production of interferon-gamma and interleukin-12 and reducing production of interleukin-10 and is essential in the pathway that leads to type I immunity. CR1 (Complement C3b/C4b Receptor 1) encodes a monomeric single-pass type I membrane glycoprotein found on erythrocytes, leukocytes, glomerular podocytes, and splenic follicular dendritic cells. This protein mediates cellular binding of particles and immune complexes that have activated complements. CD274 (CD274 Molecule; also commonly referred to as PDL1) encodes an immune inhibitory receptor ligand that is expressed by hematopoietic and nonhematopoietic cells such as T cells, B cells, and various types of tumor cells. The encoded protein is a type I transmembrane protein that has immunoglobulin V-like and C-like domains. Interaction of this ligand with its receptor inhibits T-cell activation and cytokine production. During infection or inflammation of normal tissue this interaction is important for preventing autoimmunity by maintaining homeostasis of the immune response. AIM2 (Absent in Melanoma 2) is involved in innate immune response by recognizing cytosolic double-stranded DNA and inducing caspase-1-activating inflammasome formation in macrophages; diseases associated with AIM2 include skin conditions and melanoma.

### 3.2. Synthesized Data

To investigate whether the sign average method provides a valuable summary on one gene's expression value across time (and therefore is helpful for feature selection of longitudinal gene expression data), we used observed gene expression values of the injury gene expression dataset to design two sets of simulations. Here, the expression values of each gene were further standardized to have a mean of 0 and a standard deviation of 1.

**Simulation 3.2 I.**

In Simulation I, we chose two genes (F13A1 and GSTM1) as relevant genes and then randomly included 998 other genes as noise. Denoting the expression value of gene *k* at the t^th^ time point as its symbol with a subscript of t, the probability of an injury with complication was calculated on the basis of the following logit function:

(7) logitc/u=0.57×F13A13−0.73×GSTM12+0.38×GSTM14

In this logit function, it is observed that the probability of having a complicated injury is only associated with the expression values of F13A1 at the third time point and those of GSTM1 at points 2 and 4. Furthermore, the directions of those associations are opposite. The scenario is referred to as the alternating effect case. Under this scenario, we simulated 50 datasets/replicates and used the proposed method and other relevant methods to analyze these 50 simulated datasets. Based on the calculated performance statistics given in Table 3, a comparison among the proposed methods and other relevant methods was made.

**Simulation 3.2 II.**

In Simulation II, we explored a scenario where the association presents a monotonically changed pattern; namely, the coefficients change decreasingly or increasingly over time. Again, we used F13A1 and GSTM1 as the relevant genes and randomly chose 998 of the remaining genes as noise. Denoting the expression value of gene *k* at the t^th^ time point as its symbol with a subscript of t, the corresponding logit function can be written as

(8) logitc/u=0.57×F13A11+0.67×F13A12+0.77×F13A13+0.87×F13A14+0.97×F13A15−1.02×GSTM11−0.92×GSTM12−0.82×GSTM13−0.72×GSTM14−0.62×GSTM15

This simulation setting is referred to as the monotonic effect scenario. Performance statistics were calculated and averaged for 50 replicates. The results of Simulation II are presented in Table 4.

Consistent with the results of the injury application, the methods under consideration may be roughly classified into three categories on the basis of the calculated performance statistics in Tables 3 and 4. Among them, the proposed sign average and TGDR method has the best overall performance. Since the true causal genes are known in these simulations, the ability of identifying these true relevant genes becomes another crucial index of how a feature selection algorithm performs. Although the proposed methods cannot distinguish important time points from insignificant ones or discriminate different changing patterns such as a constant or an alternating change, both methods—especially the sign average and TGDR method—identify the true causal genes with the highest frequencies and control the final model's size to a reasonable scale. Another finding is that the magnitude of an association might play a very important role in these two scenarios. Specifically, a gene with a large coefficient is more likely to be correctly identified than a gene with a small coefficient on the basis of the frequencies of these two genes being selected in the three simulations.

## 4. Conclusions

**Figure 3:**
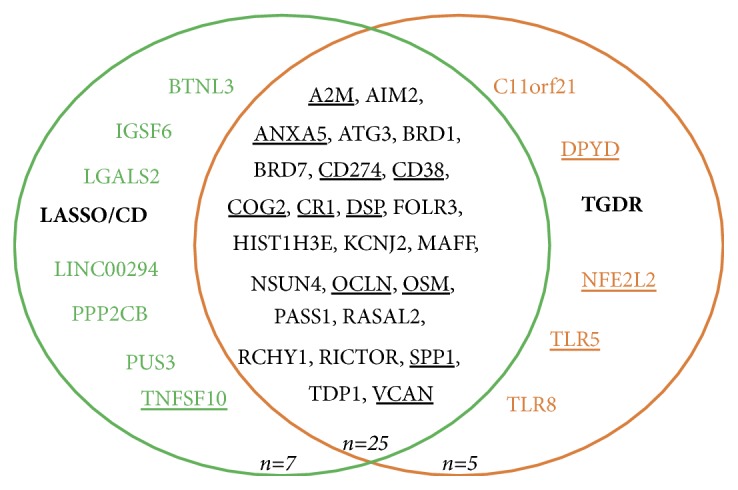
*Venn diagram illustrates the overlap of genes selected by the sign average and TGDR method and the sign average and CD method for the injury application*. Genes directly related to injury according to the Genecards database are underlined.

In this study, two optimization methods to solve a regularized regression model (the CD method and the TGDR method) were compared to investigate whether their results are comparable. A Venn diagram (Figure 3) shows the resulting gene signatures identified by the sign average and LASSO/CD method (here, the penalty function considered is LASSO) and the sign average and TGDR method. By carrying out Fisher's exact test, the corresponding p-value <2.2 × 10^−16^ indicates that these two gene lists overlap substantially (67.6%).

In terms of computing time, the TGDR method is less efficient than the CD method. The CD method took 0.205 seconds for a single run while the TGDR took 7.948 seconds for a single run on a Mac Pro laptop equipped with a 2.2 GHz dual-core processor and 16 GB RAM. The inferiority of the TGDR method regarding computing time may be due to two reasons. First, the R-codes we adapted from the meta-TGDR programming [38] do not implement any fast updating strategy. Second, the updating speed of the CD method is carried out with a call on the Fortran programming language. But implementation of the TGDR method is conducted completely in the R environment, leaving the TGDR method lagging behind the CD method. Further study on ways to update the coefficients fast and efficiently in the TGDR method is warranted.

One major contribution of this study is the proposal of using the sign average operator to integrate a gene's expression profiles across time for a specific patient into a single value. With a summary value for each gene, longitudinal data are transferred into cross-sectional data, which makes the typical feature selection algorithms plausible for longitudinal gene expression data. One criticism is that this simplification makes the crucial time points and the change pattern of expression values across time for a specific gene nonidentifiable. Nevertheless, Simulation I shows that failure to identify significant time points for individual genes does not affect the superiority of the proposed methods over other relevant algorithms.

In conclusion, summarizing genes' expression values across time using the sign average method degrades the feature selection process for longitudinal data to a conventional cross-sectional feature selection process and thus successfully conquers the longitudinal feature selection problem.

In this study, data from a microarray experiment were used to illustrate the proposed methods. However, the methods are not specific to microarray data; they can be used to analyze RNA-seq data as well. The essential steps of the proposed methods are to get a summary score for each gene (over its expression values across different time points) and then to carry out feature selection using these summary scores as predictors instead. The steps are very flexible and can be adapted to other types of gene expression data as long as the data are appropriately normalized. Specifically, for RNA-seq data, some normalized measures (e.g., transcripts per kilobase million on the log scale) would be used to quantify gene expression values.

Applying the proposed methods to one real-world dataset and two simulations, the proposed methods, especially for the sign average and TGDR method, present superiority over other relevant algorithms. Therefore, the proposed methods are highly recommended.

## Figures and Tables

**Table 1:** Overview of methods under consideration.

Method	GX	Pseudo-	If using pseudo-genes, which summary score is used
	Values^1^	Genes^2^	
Sign Avg & LASSO/CD		√	Sign average of a gene's expression over time.

Sign Avg &TGDR		√	Sign average of a gene's expression over time.

Mean & LASSO/CD		√	Mean of a gene's expression value over time.

Mean & TGDR		√	Mean of a gene's expression value over time.

Median & LASSO/CD		√	Median of a gene's expression value over time.

Median & TGDR		√	Median of a gene's expression value over time.

PC1 & LASSO/CD		√	First principal component of a gene's expression value over time

PC1 & TGDR		√	First principal component of a gene's expression over time.

EDGE	√		

limma	√		

LASSO/CD separately	√		

TGDR separately	√		

glmmLASSO	√		

^1^ GX values represent actual expression values.

^2^ Pseudogenes are generated to summarize expression values across time.

**Table 2:** Performance of the proposed method on the traumatic injury application and comparison with other methods.

Method		Size	Rand Index	Test Set		
				Error rate	BCM^1^	AUPR^2^
Proposed methods	Sign Avg. & LASSO/CD^3^	32	19.58%	**0.351**	**0.605**	0.626
	Sign Avg. & TGDR^4^	30	25.21%	0.378	0.590	**0.662**

Existing methods	limma	47	21.40%	0.432	0.542	0.628
	EDGE	453	13.67%	0.432	0.543	0.622
	glmmLASSO	8	34.99%	0.432	0.519	0.532
	LASSO/CD separately^5^	28	13.59%	0.486	0.498	0.508
	TGDR separately^6^	133	22.58%	0.378	0.520	0.579
	Mean & LASSO/CD^7^	29	17.95%	0.405	0.536	0.560

Using other summary scores	Mean & TGDR^8^	36	27.37%	0.405	0.562	0.617
	Median & LASSO/CD^9^	22	7.76%	0.351	0.543	0.617
	Median & TGDR^10^	43	18.58%	0.405	0.578	0.626
	PC1 & LASSO/CD^11^	3	13.59%	0.405	0.504	0.541
	PC1 & TGDR^12^	29	32.68%	0.432	0.539	0.548

^**1**^BCM captures the average confidence that a sample belongs to class i when it indeed belongs to that class;

^**2**^AUPR is the average of AUPR_k_ for each class and it captures the ability of correctly ranking the samples known to belong in a given class;

^**3**^Sign Avg. & LASSO/CD: pseudo genes were obtained by calculating the sign average of a gene's expression values across time, and the feature selection method is LASSO in which the optimization method used is coordinate descent;

^4^Sign Avg. & TGDR: pseudo genes were obtained by calculating the sign average of a gene's expression values across time, and the feature selection/optimization method is threshold gradient descent regularization;

^5^LASSO/CD separately: separate LASSO models were trained at individual time points; the optimization method is CD;

^6^TGDR separately: separate TGDR models were trained at individual time points; the optimization method is TGDR;

^7^Mean & LASSO/CD: pseudo genes were obtained by calculating the average of a gene's expression values across time, and the optimization method is CD;

^8^Mean & TGDR: pseudo genes were obtained by calculating the average of a gene's expression values across time, and the optimization method is TGDR;

^9^Median & LASSO/CD: pseudo genes were obtained by calculating the median of a gene's expression values across time, and the optimization method is CD;

^10^Median & TGDR: pseudo genes were obtained by calculating the median of a gene's expression values across time, and the optimization method is TGDR;

^11^PC1 & LASSO/CD: pseudo genes obtained by calculating the first principal component of a gene's expression values across time, and the optimization method is CD;

^12^PC1 & TGDR: pseudo genes were obtained by calculating the first principal component of a gene's expression values across time, and the optimization method is TGDR.

**Table 3:** Performance of the proposed methods and other relevant methods on Simulation I.

Method	Size	Rand	F13A1	GSTM1	Error rate^1^	BCM^2^	AUPR^3^
		(%)	(%)	(%)	(%)		
Sign Avg. & LASSO/CD^4^	5.52	13.78	70	10	22.97	0.582	0.873

Sign Avg. & TGDR^5^	16.76	8.12	88	100	6.77	0.724	0.987

EDGE*∗*	20	3.85	16	0	10.80	0.719	0.936

limma	6.04	11.72	8	100	16.17	0.707	0.908

LASSO/CD separately^6^	4.65	29.17	36	40	30.00	0.527	0.924

TGDR separately^7^	32.26	5.30	100	100	19.27	0.611	0.991

glmmLASSO	114.06	3.05	0	0	36.40	0.519	0.571

*∗*Using q-value as the cutoff, EDGE selects all 1,000 genes as significant. We used the 20 most significant genes instead. ^1^Error rate = (false positives + false negatives)/(sample size).

^2^BCM captures the average confidence that a sample belongs to class i when it indeed belongs to that class.

^3^AUPR is computed as the average of the AUPR_k_ for each class and captures the ability of correctly ranking the samples known to belong in a given class.

^4^Sign Avg. & LASSO/CD: Pseudogenes were obtained by calculating the sign average of a gene's expression values across time; the optimization method is coordinated descent.

^5^Sign Avg. & TGDR: Pseudogenes were obtained by calculating the sign average of a gene's expression values across time; the optimization method is threshold gradient descent regularization.

^6^LASSO/CD separately: separate LASSO models were trained at individual time points; the optimization method is CD.

^7^TGDR separately: separate TGDR models were trained at individual time points; the optimization method is TGDR.

**Table 4:** Performance of the proposed methods and other relevant methods on Simulation II.

Method	Size	Rand	F13A1	GSTM1	Error rate^1^	BCM^2^	AUPR^3^
		(%)	(%)	(%)	(%)		
Sign Avg. & LASSO/CD^4^	13.82	10.03	100	96	1.27	0.854	0.994
Sign Avg. & TGDR^5^	9.92	14.78	100	96	3.33	0.841	0.993
EDGE*∗*	20	2.72	0	0	7.37	0.755	0.973
limma	8.9	9.75	0	100	5.23	0.809	0.981
LASSO/CD separately^6^	15.88	8.81	98	100	6.60	0.668	0.982
TGDR separately^7^	75.48	3.38	100	100	4.47	0.714	0.991
glmmLASSO	63.52	1.63	4	8	46.77	0.510	0.551

*∗*Using q-value as the cutoff, EDGE selects all 1,000 genes as significant. We used the 20 most significant genes instead. ^1^Error rate = (false positives + false negatives)/(sample size).

^2^BCM captures the average confidence that a sample belongs to class i when it indeed belongs to that class.

^3^AUPR is computed as the average of the AUPR_k_ for each class and captures the ability of correctly ranking the samples known to belong in a given class.

^4^Sign Avg. & LASSO/CD: Pseudogenes were obtained by calculating the sign average of a gene's expression values across time; the optimization method is coordinated descent.

^5^Sign Avg. & TGDR: Pseudogenes were obtained by calculating the sign average of a gene's expression values across time; the optimization method is threshold gradient descent regularization.

^6^LASSO/CD separately: separate LASSO models were trained at individual time points; the optimization method is CD.

^7^TGDR separately: separate TGDR models were trained at individual time points; the optimization method is TGDR.

## Data Availability

Data were retrieved from the Gene Expression Omnibus repository (http://www.ncbi.nlm.nih.gov/geo/). The accession number is GSE36809.

## Supplementary Materials

Supplementary Materials Supplementary File 1: R codes for the proposed method (the sign average and TGDR method).
