# Supplementary material for: Feature Selection for Longitudinal Data by Using Sign Averages to Summarize Gene Expression Values over Time
Source: Biomed Res Int. 2019 Mar 19;2019:1724898. doi: 10.1155/2019/1724898 (PMC6444255; doi:10.1155/2019/1724898)
Supplement: Supplementary Materials — Supplementary File 1: R codes for the proposed method (the sign average and TGDR method). [file 1724898.f1.docx]

# A function to get the estimated signs for the sign average method

get_sign<-function(exp.train, meta.train, obj="class"){

sign<-NULL

time.point=unique(meta.train$time)

if (obj=="class"){

library(limma)

for (k in 1:length(time.point)){

exp.train.t<-exp.train[,meta.train$time==k]

design<-model.matrix(~as.factor(meta.train$group[meta.train$time==k]))

fit.try<-lmFit(exp.train.t, design)

sign<-cbind(sign, ifelse(fit.try$coef[,2]>0, 1, -1))

}

}

return(sign)

}

# A function to get the sign averages for all genes in the training set.

# If there is a test set, get the sign averages for all genes in the test set as well.

sign_ave2<-function(exp.train, meta.train, obj="class", exp.test=NULL, meta.test=NULL){

sign<-get_sign(exp.train, meta.train, obj="class")

sign.ave<-matrix(0, nrow=dim(exp.train)[1], ncol=length(unique(meta.train$id)))

for (j in 1:length(unique(meta.train$id))){

exp.train.junk<-exp.train[,meta.train$id==unique(meta.train$id)[j]]

ncol<-dim(exp.train.junk)[2]

sign.sel<-sign[,1:ncol]

sign.ave[,j]<-apply(exp.train.junk*sign.sel, 1, mean)

}

if(length(exp.test)!=0){

sign.ave.test<-matrix(0, nrow=dim(exp.test)[1], ncol=length(unique(meta.test$id)))

for (k in 1:length(unique(meta.test$id))){

exp.test.junk<-exp.test[,meta.test$id==unique(meta.test$id)[k]]

ncol<-dim(exp.test.junk)[2]

sign.sel<-sign[,1:ncol]

sign.ave.test[,k]<-apply(exp.test.junk*sign.sel, 1, mean)

}

return(list(sign.ave, sign.ave.test))

}else{

return(sign.ave)

}

}

#The TGDR function

#here, all.y represents labels aug.x represents a constant term +expression values profiles

# opt.s: the number of steps to run, a tuning parameter whose value is determined by using cross-validations

# tau: the other tuning parameter, its default value is 1 for high-dimensional data

# increase: a small valued increment.

tgdr.est<-function (all.y, aug.x, opt.s, increase = 0.01, tau = 1){
    num.cov <- nrow(aug.x) - 1
    gradient.mat <- matrix(0, (num.cov + 1), 1)
    beta.mat <- matrix(0, (num.cov + 1), 1)
    all.x<-aug.x[-1,]
    aug.z.dat <- aug.x
    indicator <- all.y
    beta.out <- NULL
    for (s in 1:opt.s) {
        work.z.dat <- aug.z.dat
        work.mat <- matrix(0, nrow(work.z.dat), ncol(work.z.dat))
        for (mm in 1:ncol(work.z.dat)) work.mat[, mm] <- work.z.dat[,
            mm]
        work.beta <- beta.mat
        work.y <- indicator
        beta.z <- t(work.mat) %*% work.beta
        beta.p <- 1/(1 + exp(-beta.z))
        term.1 <- work.y/beta.p - (1 - work.y)/(1 - beta.p)
        term.2 <- exp(beta.z)/((1 + exp(beta.z))^2)
        work.gradient <- (t(term.1 * term.2)) %*% t(work.mat)
        gradient.mat <- work.gradient
        max.gradient <- max(gradient.mat)
        gradient <- gradient.mat
        f.v <- ifelse(abs(gradient.mat) >= abs(tau * max.gradient),
            1, 0)
        f.v[1]<-1    
        g.v <- gradient * f.v
        beta.mat <- beta.mat + t(increase * g.v)
        beta.out <- cbind(beta.out, beta.mat)
    }
    rownames(beta.out[-1, ]) <- rownames(all.x)
    beta.sum <- apply(abs(beta.mat), 1, sum)
    size <- length(beta.sum[beta.sum != 0])
    samplesize <- length(all.y)
    score <- rep(0, samplesize)
    for (i in 1:samplesize) {
        x <- aug.z.dat[, i]
        score[i] <- sum(x * beta.mat)
    }
    cut <- sort(score, decreasing = TRUE)[sum(all.y)]
    pred <- rep(0, samplesize)
    for (i in 1:samplesize) {
        pred[i] <- ifelse(score[i] >= cut, 1, 0)
    }
    return(list(pred = pred, beta = beta.out))
}
